# Microbial Reprogramming Inhibits Western Diet-Associated Obesity

**DOI:** 10.1371/journal.pone.0068596

**Published:** 2013-07-10

**Authors:** Theofilos Poutahidis, Markus Kleinewietfeld, Christopher Smillie, Tatiana Levkovich, Alison Perrotta, Siddheshvar Bhela, Bernard J. Varian, Yassin M. Ibrahim, Jessica R. Lakritz, Sean M. Kearney, Antonis Chatzigiagkos, David A. Hafler, Eric J. Alm, Susan E. Erdman

**Affiliations:** 1 Division of Comparative Medicine, Massachusetts Institute of Technology, Cambridge, Massachusetts, United States of America; 2 Laboratory of Pathology, Faculty of Veterinary Medicine, Aristotle University of Thessaloniki, Thessaloniki, Greece; 3 Departments of Neurology and Immunobiology, Yale School of Medicine, New Haven, Connecticut, United States of America; 4 Broad Institute, Massachusetts Institute of Technology and Harvard University, Cambridge, Massachusetts, United States of America; 5 Civil and Environmental Engineering, Massachusetts Institute of Technology, Cambridge, Massachusetts, United States of America; 6 Biological Engineering, Massachusetts Institute of Technology, Cambridge, Massachusetts, United States of America; National Jewish Health and University of Colorado School of Medicine, United States of America

## Abstract

A recent epidemiological study showed that eating ‘fast food’ items such as potato chips increased likelihood of obesity, whereas eating yogurt prevented age-associated weight gain in humans. It was demonstrated previously in animal models of obesity that the immune system plays a critical role in this process. Here we examined human subjects and mouse models consuming Westernized ‘fast food’ diet, and found CD4^+^ T helper (Th)17-biased immunity and changes in microbial communities and abdominal fat with obesity after eating the Western chow. In striking contrast, eating probiotic yogurt together with Western chow inhibited age-associated weight gain. We went on to test whether a bacteria found in yogurt may serve to lessen fat pathology by using purified *Lactobacillus reuteri* ATCC 6475 in drinking water. Surprisingly, we discovered that oral *L. reuteri* therapy alone was sufficient to change the pro-inflammatory immune cell profile and prevent abdominal fat pathology and age-associated weight gain in mice regardless of their baseline diet. These beneficial microbe effects were transferable into naïve recipient animals by purified CD4^+^ T cells alone. Specifically, bacterial effects depended upon active immune tolerance by induction of Foxp3^+^ regulatory T cells (Treg) and interleukin (Il)-10, without significantly changing the gut microbial ecology or reducing *ad libitum* caloric intake. Our finding that microbial targeting restored CD4^+^ T cell balance and yielded significantly leaner animals regardless of their dietary ‘fast food’ indiscretions suggests population-based approaches for weight management and enhancing public health in industrialized societies.

## Introduction

The risk of developing obesity rises with a Westernized lifestyle. In industrialized and developing countries obesity contributes to increased mortality by predisposing to serious pathological conditions such as type 2 diabetes, cardiovascular disease, fatty liver, arthritis, asthma, and neoplasia [1]–[2]. Clinical and experimental data suggest that the white adipose tissue of obese organisms is in a low-grade, persistent state of chronic inflammation that exerts adverse systemic effects [2]–[3]. The most prominent inflammatory cell type of the obesity-associated inflammation is the adipose tissue macrophage. Macrophages are recruited and surround dead adipocytes, thus creating the so-called crown-like structures (CLS). These cells along with hypertrophic adipocytes are thought to be the key cells initiating the unique subclinical pro-inflammatory signaling cascade encountered in obesity [2], [4]–[5]. Macrophages, B and T lymphocytes, and up-regulated pro-inflammatory cytokines including TNF-α, IL-1, IL-6, IL-17, and monocyte chemoattractant protein-1 (MCP-1) have been reported to contribute to obesity-associated pathologies. In parallel, regulatory T cells down-regulate host inflammatory responses [2]–[3], [6]–[10].

It is well documented that “fast food” with high fat and salt content at relatively low cost is a major cause of the obesity epidemic in Western societies. Recent epidemiological research shows while dietary ‘fast food’ contributes to obesity, eating yogurt surprisingly prevents age-associated weight gain, though the mechanism is unknown. It has been thought that slenderizing outcomes of yogurt are due to a probiotic bacteria-mediated mechanism [1]. Dietary probiotic consumption alters gut microbiota and may be an effective strategy not only for weight loss but also for preventing weight regain after loss [11]–[14]. Furthermore, alterations in the composition of gut microbiota may affect not only gut health but also distant tissues and overall health and longevity via immune-mediated mechanisms [15]–[20].

Using a mouse model of obesity we found that purified probiotic organisms alone prevented weight gain, and these protective effects were irrespective of the baseline diet. We show that this effect could be isolated to a single purified probiotic microbe, namely *Lactobacillus reuteri*. Importantly, eating *L. reuteri* bacteria acted without changing the existing gastrointestinal (GI) microbial composition in stool or level of calorie consumption; instead, the slenderizing microbial mechanism involved bacteria-triggered changes in the host immune system composition. The effect was in particular dependent on CD4^+^ T cells and the presence of anti-inflammatory Il-10, as Il-10 deficient animals were resistant to *L. reuteri*-induced effects. Adoptive transfer of purified Il-10-competent *L. reuteri*-induced Foxp3^+^ Treg cells was sufficient to rescue fat pathology and lessen body fat in naïve recipient animals. These data provide a mechanism whereby simple dietary manipulation can have major health impact, highlighting the utility of directly harnessing bacteria for public health initiatives.

## Results

### Eating Westernized ‘Fast Food’ Style Diet Restructures the Gut Microbiome and Accelerates Age-associated Obesity in Mice

**Figure 1:**
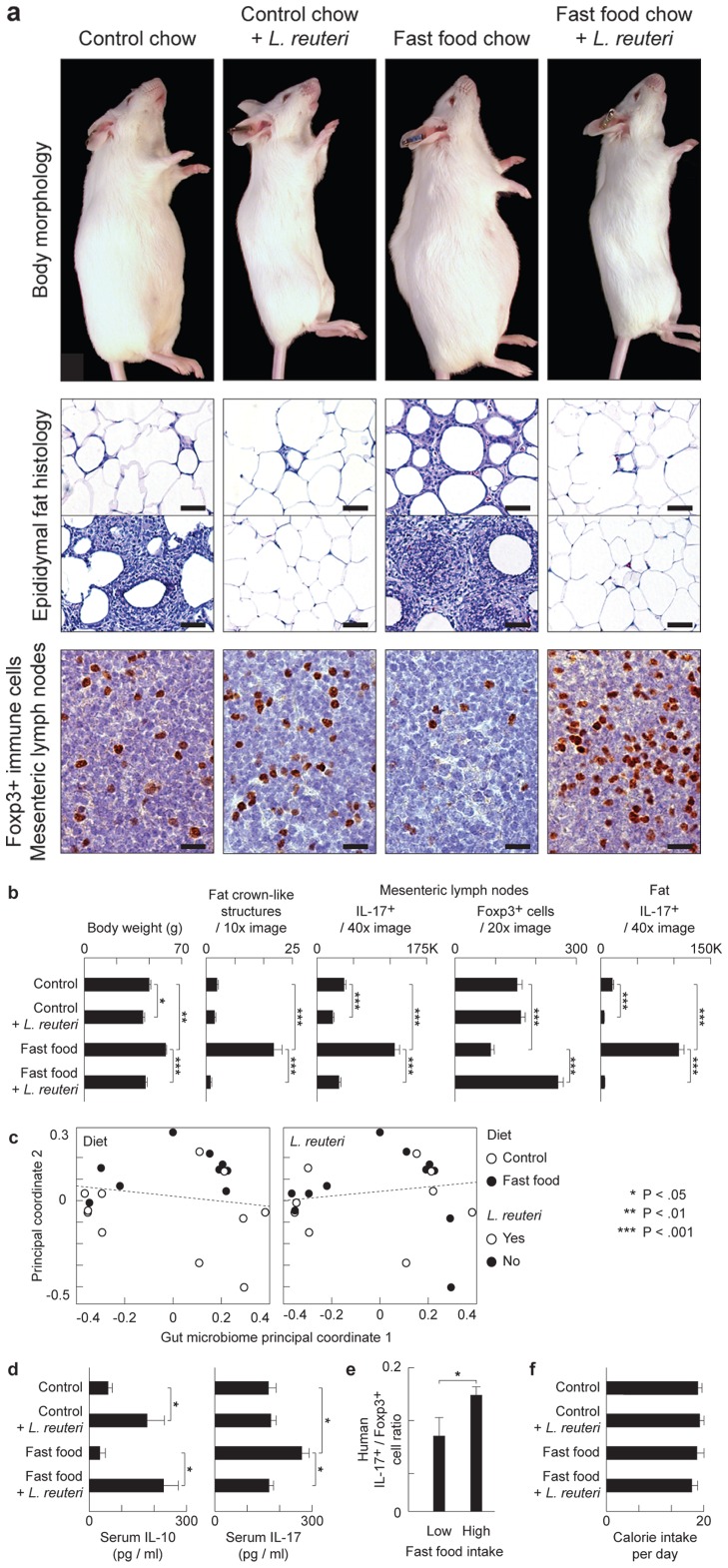
Eating probiotics blocks a gut microbiota-immunity-obesity axis. *L. reuteri* protects mice from Western diet-associated obesity. Data are shown in male outbred Swiss mice at the age of 5 months. Numerous crown-like structures (CLS) caused by adipocyte death-related inflammation, and focal pyogranulomatous inflammation (PGI) arise in abdominal fat of ‘fast food’-fed but not probiotic-fed animals. Probiotics increase anti-inflammatory Foxp3+ regulatory (Treg) cells and reduce pro-inflammatory Il17 protein to restore immune balance coinciding with a slender physique (**a and b**), without restructuring GI microbial communities (**c**). In the same mice, serum cytokine analysis shows that the pro-inflammatory Il-17-associated effect of obesity is systemic, and that *L. reuteri* negates this effect up-regulating the anti-inflammatory cytokine Il-10 (**d**). Humans frequently eating ‘fast food’ also show an elevated ratio of pro-inflammatory IL17+/anti-inflammatory Foxp3+ Treg in peripheral blood cells compared to subjects never eating ‘fast food’ (**e**)**.** Probiotic-consuming slim mice chose similar calories when compared with obese animals, regardless of baseline diet, highlighting potential for translational medicine (**f**). Fat histology: Hematoxylin and eosin, Bars = 50 µm; MLN Immunohistochemistry: Diaminobenzidine chromogen, hematoxylin counterstain, Bars = 8.3 µm.

Knowing that eating ‘fast food’ contributes to age-associated weight gain in humans, we first used animal models to test specific roles for diet and the gastrointestinal (GI) tract microbiome in obesity. Genetically outbred Swiss mice were fed an *ad libitum* diet of Westernized chow mimicking typical human ‘fast food’ diets that are high in fat and sugar, and low in fiber and vitamins B and D, that lead to age-associated obesity (Figure 1a). Abdominal fat examined histologically at five months of age revealed increased crown-like structures (CLS) (Figs. 1a and 1b) and a type of pyogranulomatous inflammation characteristic of obesity in humans [2]. Although both genders exhibited significant increases in fat pathology after Western chow, both CLS and pyogranulomatous inflammation lesions were more pronounced in male mice than in female mice when examined at five months of age (Fig. S1). Gastrointestinal tract microbial communities in mice changed within weeks after beginning Western diet formula (Fig. 1c; Fig. S1), showing that altered gut microbes may be associated with weight gain and obesity [1], [11].

### Subjects Dining on Westernized ‘Fast Food’-style Diet Exhibit Th17-biased Immunity

It is well established that intestinal microbes modulate host health through activities of CD4^+^ T cells [21]–[22], at least in part through Il-6-dependent reciprocal functions of anti-inflammatory Foxp3^+^ Treg cells and pro-inflammatory Th17 cells [6], [23]. Thus, we examined T cell subpopulations and found that obese mice eating Western chow had increased frequencies of Il-17 expressing cells which is in line with a previous report [6] (Fig. 1b and 1d). Importantly, when examining peripheral blood of human subjects frequently dining on ‘fast food’ we found a similar pro-inflammatory Th17-biased profile (Fig. 1e).

### Dietary Supplementation with Probiotic Yogurt Inhibits Obesity Due to Westernized ‘Fast Food’ Style Diet

Recent epidemiological research shows eating “yogurt” prevents age-associated weight gain in humans [1]. To examine whether this epidemiologic observation could be modeled in genetically outbred experimental animals, and as a prelude to testing isolated microbes, we first examined the effect of a commercially available probiotic yogurt by feeding 0.8ml/mouse thrice weekly to Swiss mice eating either control or Westernized diets. Surprisingly, we discovered that feeding of probiotic yogurt together with either control chow (N = 5 mice/group; body weight of mice eating control diet = 37.42±4.711g versus control diet+yogurt = 24.9±4.995, p<0.05), or with ‘fast food’ style chow (fast food diet = 42.39±7.455 versus fast food diet+yogurt = 28.08±0.732, p<0.001), entirely inhibited the age-associated fat pathology accumulation and body weight gain when examined upon necropsy at five months of age. Differences in body weight were attributable at least in part to intra-abdominal fat, which was significantly reduced in mice eating the probiotic yogurt (intra-abdominal fat of mice eating control diet = 1.692±0.9036 g versus control diet+yogurt = 0.3067±0.1684, p<0.05; fast food diet = 3.06±0.9737 versus fast food diet+yogurt = 0.5054±0.2536, p<0.05). These data showed that probiotic yogurt yielded significantly leaner animals regardless of dietary ‘fast food’.

### Feeding of Purified *Lactobacillus reuteri* was Sufficient to Inhibit Western Diet Obesity

**Figure 2:**
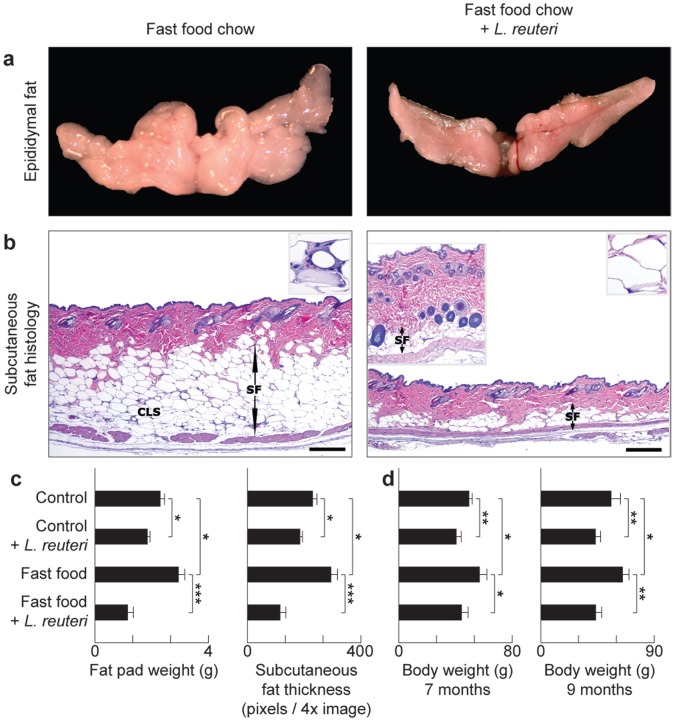
Dietary supplementation with L. reuteri protects from age-associated Western diet obesity. Specifically, the abdominal (epididymal) fat mass is significantly reduced in probiotic-consuming Swiss mice (**a**). The slenderizing effect of *L. reuteri* is also observed in the subcutaneous fat depot. The subcutaneous fat layer (SF) is significantly thicker and has many CLS (inset) in “fast food”-fed mice in contrast to mice eating the same diet and *L. reuteri*. There is thicker dermis and increased subcutaneous hair follicle profiles in the left inset of the “fast food”+probiotic skin image (**b**). Fad pad weight and subcutaneous fat thickness histomorphometric analyses show that probiotics protect from age-associated obesity irrespective of baseline diet (**c**). Eating probiotics benefits aged Swiss mice as well as the young animals, evident here from the body weight analysis of 7- and 9-months-old male and female mice (**d**). Skin histology: Hematoxylin and eosin, Bars = 250 µm.

**Figure 3:**
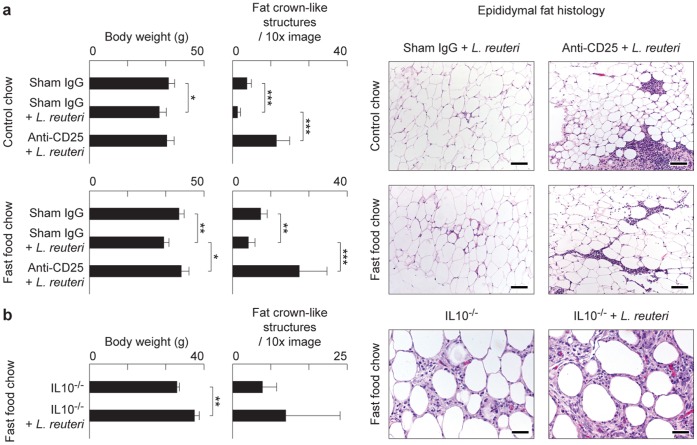
Mice exhibit an Interleukin 10-dependent Treg cell-mediated gut microbiota-immunity-obesity axis. Genetically-inbred C57BL/6 strain mice similarly benefit from probiotic protection against Western diet-associated obesity and fat pathology, including CLS and focal pyogranulomatous inflammation as shown here in males at 5 months of age (**a**). *Interleukin (Il) 10*-deficient C57BL/6 male mice eating Westernized chow failed to benefit from oral *L. reuteri* supplementation. Mice from both experimental groups were obese and had increased CLS that were often seen coalescing to form focally diffuse areas of adipocyte necrosis (**b**). Fat histology: Hematoxylin and eosin. Bars = 250 µm (a) and 100 µm (b).

To examine whether the effect of the yogurt was due to probiotic bacteria, or instead other compounds such as extra protein or vitamin D supplied in yogurt, we fed mice purified probiotic organism *Lactobacillus reuteri* ATCC 6475 cultivated as described elsewhere [24] using a dosage of 3.5×10^5^ organisms/mouse/day in drinking water. Mice received the organisms as above in their drinking water starting at age 8 weeks and continuing for three months throughout the study until necropsy at age = five months. We found probiotic bacteria alone were sufficient for the slenderizing effect and entirely blocked development of abdominal fat pathology arising from *ad libitum* feeding of Western ‘fast food’ chow (Figs. 1a and 1b). Abdominal fat (Fig. 2a) and subcutaneous fat (Fig. 2b) accumulations were significantly reduced in Swiss mice eating purified *L. reuteri* in combination with either control or Western diet (Fig. 2a–c). This was not a generic attribute of bacteria added to the drinking water, as mice consuming 3.5×10^5^ *Escherichia coli* K12 organisms/mouse/day in drinking water did not develop the slender physique (Fig. S1). This protection from age-associated weight gain was sustained as evidenced by older Swiss mice at seven-months and nine-months of age (Fig. 2d) while consuming 3.5×10^5^ *L. reuteri* organisms daily in their drinking water also starting at the age of 8 weeks. Similar outcomes were also achieved in inbred C57BL/6 strain mice eating special diets with or without *L. reuteri* supplementation (Figs. 3a). Taken together, these data demonstrate that purified lactic acid bacteria, in this case *L. reuteri* ATCC 6475 organisms alone, are sufficient for the anti-obesity effects of eating a probiotic yogurt formulation.

### Eating *L. reuteri* Daily did not Change the Existing Gut Microbiome

Knowing that eating probiotics made mice slim, we next tested whether purified probiotic *L. reuteri* may act to change the microbial ecology in the gut. Using paired-end Illumina sequencing targeting the V4 region of the 16S rRNA gene on animal stools, it was found that daily intake of probiotic organisms did *not* change the existing gut microbiome profile (Fig. 1c). Thus, animals eating *L. reuteri* had a GI tract microbiome output that was not significantly different from their matched diet counterparts eating either regular chow or Western diet. Although probiotics didn’t restructure resident microbiota communities, a pre-existing diverse microbial community was required for optimal slenderizing effects as illustrated by the fact that mice raised under germ-free conditions, and then fed *L reuteri* under general housing conditions, fail to benefit from eating probiotic organisms (Fig. S1h).

### Probiotic Bacteria-consuming Slim Mice Chose Similar Calories as Obese Animals

Another possible explanation for significantly slimmer physiques after eating *L. reuteri* was reduced caloric intake when eating probiotic bacteria. In order to test this, we calculated daily ‘free choice’ consumption of mouse chow in animals with *L reuteri* bacteria added to their drinking water *versus* regular water controls. We found that *L reuteri*-consuming slim mice chose similar calories to those of the regular-water drinking obese animals (Fig. 1f), in spite of large (p<0.001) body weight discrepancies.

### Probiotic Microbes Inhibit Fat Pathology by an IL-10-dependent Mechanism

Recognizing that eating probiotics made mice thin without restructuring their microbial communities or reducing food intake, we hypothesized that probiotic organisms may protect from obesity by up-regulating anti-inflammatory immune activities; in particular, levels of anti-inflammatory cytokine IL-10. This reasoning was based upon data that interleukin-10 is pivotal in mounting immune tolerance to microbes along intestinal mucosal interfaces [25]–[26]. In support of this concept, it was found that the Swiss mice eating *L. reuteri* exhibited higher levels of IL-10 protein in serum than matched control mice (Fig. 1d). To test this further, we examined C57BL/6 strain mice with deletion of the *Il-10* gene and thus entirely lacking IL-10. Importantly, we found that *Il-10*-null animals eating Western chow with yogurt or Western chow with *L. reuteri* failed to benefit from probiotic bacteria and instead became obese (Fig. 3b). Likewise, C57BL/6 Rag2-deficient mice (that entirely lack functional lymphocytes) eating Westernized diets were unprotected from obesity after eating *L. reuteri* (N = 7 mice/group; body weight of mice eating Western diet = 42.94±1.19 g versus Western diet+*L reuteri* = 41.78±1.45, p = 0.1 not significant). Taken together these data indicated that probiotic microbes acted to inhibit fat pathology by an IL-10-dependent adaptive immune cell mechanism.

### Probiotic-triggered Protection from Fat Pathology is Transferable to Naïve Hosts via Purified CD4^+^ T Cells

**Figure 4:**
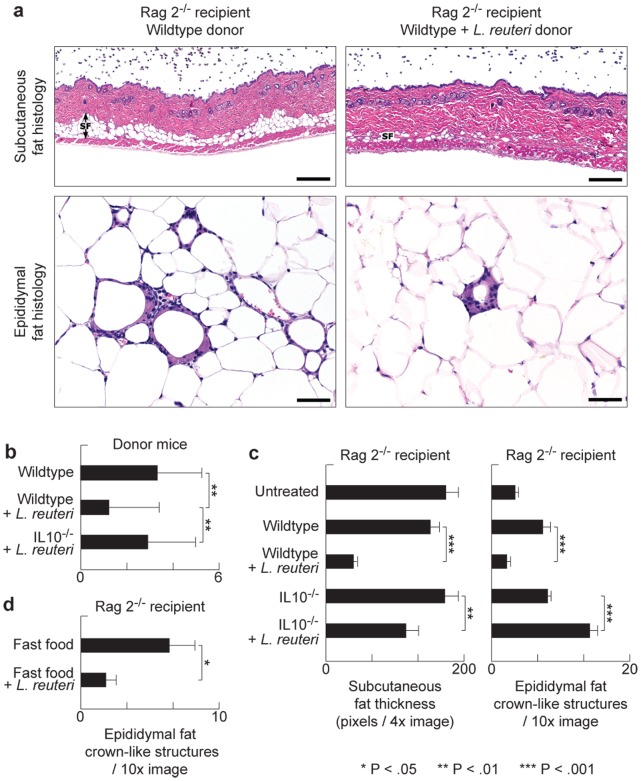
Diet-associated fat pathology is transferable to naïve animals using purified T-lymphocytes. Adoptive transfer of purified CD4+ cells from C57BL/6 *wild type* donor mice eating probiotics into C57BL/6 *Rag2*-null mice was sufficient to significantly reduce recipient body fat depots such as subcutaneous fat, as well as ameliorate abdominal fat pathology (**a**). Control diet-fed mice used as donors for lymphocyte transfer experiments showed IL10-dependent *L.reuteri* benefits including significantly less CLS in their abdominal fat (**b**). This immune-mediated protection requires Il10, as adoptive transfer of CD4^+^ cells from probiotic-fed Il10-deficient donors did not protect the recipient mice from obesity and associated fat pathology (**c**)**.** Purified wild type CD4^+^ FoxP3^+^ Treg cells from mice eating *L. reuteri* were sufficient for beneficial effects in *Rag2*-null recipient mice rescuing them from obesity-associated pathology (**d**). Skin and fat histology: Hematoxylin and eosin. Bars skin = 250 µm, edididymal fat = 50 µm.

Based upon our earlier work [27], and that of others [7], [28]–[30], we postulated that probiotic organisms such as *L reuteri* protected from obesity by IL-10-mediated induction of lymphocytes [21]–[22]. To test whether probiotic-triggered lymphocytes were sufficient for reduced fat pathology we used adoptive transfer of purified CD4^+^ T cells into naïve syngeneic C57BL/6 Rag2-deficient mice. For these experiments, cell donors were fed supplementary probiotic yogurt or ate only baseline diets, and exhibited fat pathology typical for that treatment. We found that Rag2-null recipients of cells from donor mice eating the probiotic bacteria had reduced abdominal fat pathology when compared with recipients of cells from untreated control mice (Fig. 4). Moreover, subcutaneous fat was significantly reduced in regular-chow Rag2-knockout recipients of cells from *L reuteri*- consuming mice (Fig. 4a). Importantly, these protective effects required IL-10-competency in cell donor mice (Fig. 4b–c). These data showed that the microbe-imbued protection from obesity resided in IL-10-dependent functions of CD4^+^ T cells, confirming the findings of Feuerer et al (2009) who observed that protective IL-10-dependent Treg cells are associated inversely with adiposity [31].

### Feeding of Purified *L. reuteri* Restores Foxp3^+^ Treg/Th17 Balance

Based upon earlier work by DiGiacinto et al (2005), we postulated that probiotic organisms protect from obesity by IL-10-mediated induction of anti-inflammatory Treg cells [21]–[22], [28]. We tested whether feeding of *L. reuteri* may restore host immunity from a diet-induced pro-inflammatory Th17 bias towards a beneficial anti-inflammatory Treg cell dominated immunity. We found that slim mice eating Westernized chow *plus* probiotic *L. reuteri* showed significantly increased anti-inflammatory Foxp3^+^ Treg cells and also lower levels of IL-17A protein within abdominal lymph tissues when compared with mice feeding on Westernized ‘fast food’–style chow alone (Figs. 1a and 1b). Further, humans frequently eating ‘fast food’ displayed a pro-inflammatory Th17-dominant phenotype when compared with control subjects who exhibited more Foxp3^+^ cells and fewer Th17 cells in the peripheral blood (Fig. 1e). These data are in line with an earlier study showing the importance of anti-inflammatory Treg for prevention of adiposity and insulin resistance in mouse models [31]. Thus feeding *L. reuteri* specifically induced Foxp3^+^ Treg cells and restored the Treg/Th17 balance observed in lean animals.

### *L. reuteri* Induced Effects are Dependent on Active Immune Regulation by Foxp3^+^ Treg Cells

**Figure 5:**
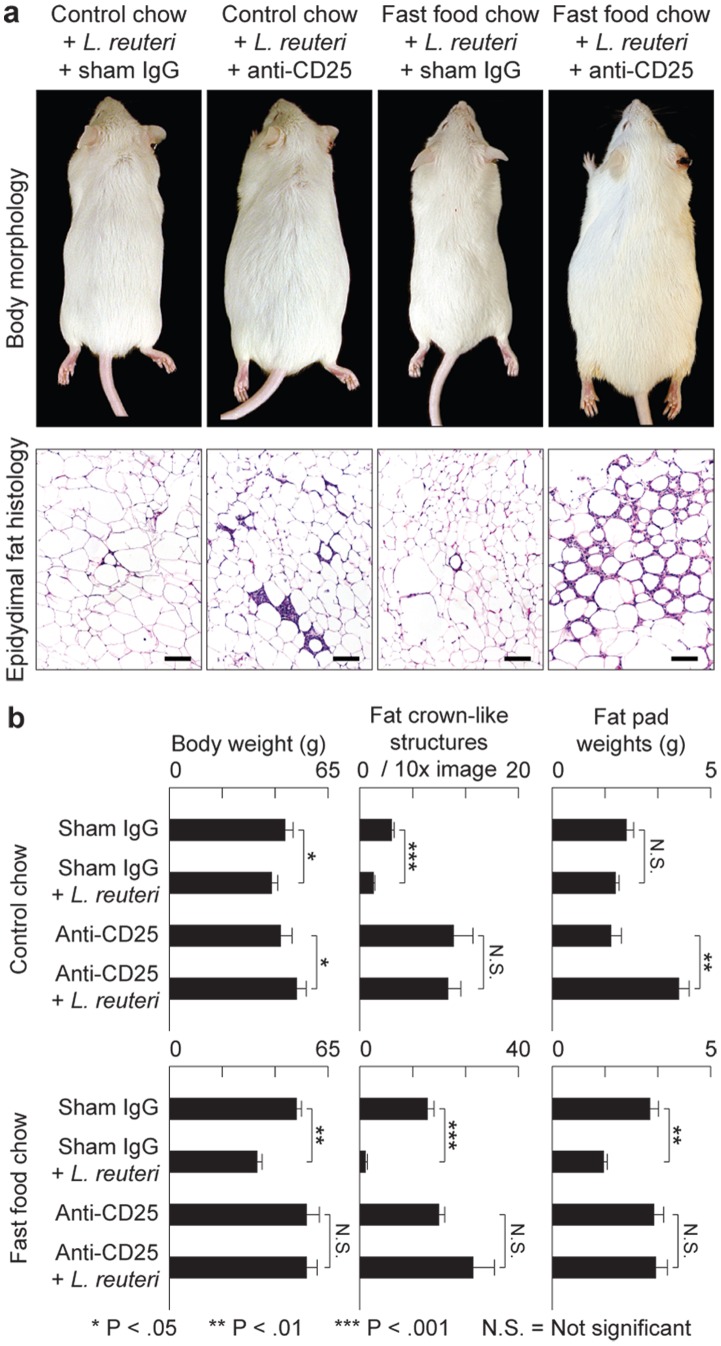
Anti-obesity protection of oral probiotics in outbred Swiss mice requires CD25^+^ immune cells. Depletion of CD4^+^CD25^+^ Treg cells entirely inhibits probiotic-induced protection from age-associated obesity and abdominal fat pathology (**a**). Probiotics protect from weight gain unless mice were simultaneously treated with anti-CD25 antibody, in which case animals rapidly became obese. Frequency of prototype crown-like structures was increased in abdominal fat after depletion of CD25^+^ cells but not in sham IgG-treated control animals (**b**). Fat histology: Hematoxylin and eosin. Bars = 100 µm.

**Figure 6:**
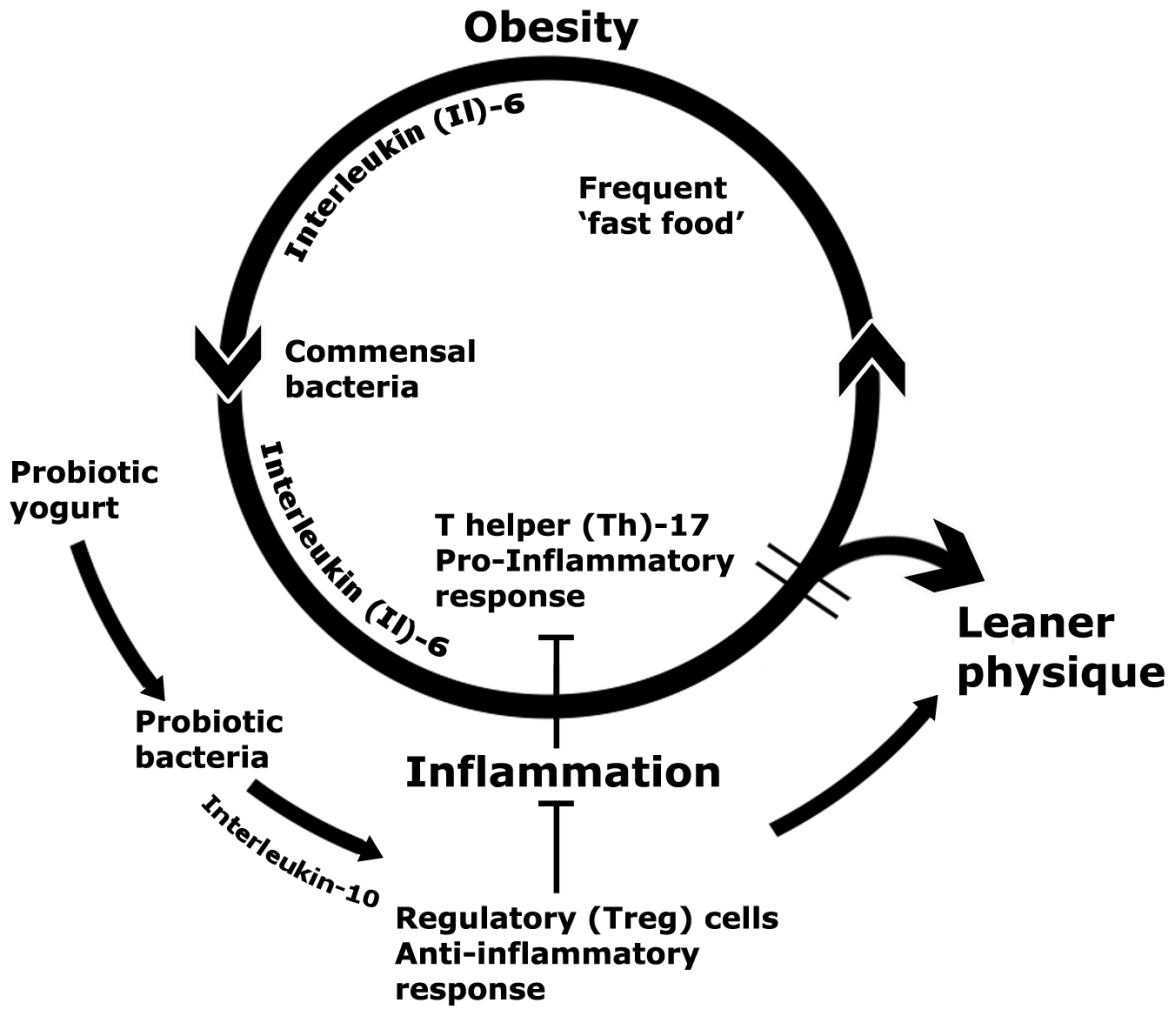
Proposed mechanistic overview. In order to explain the ‘fast food’ *versus* ‘yogurt’ age-associated weight disparity observed in human subjects, we propose that Western-style dietary habits alter gut microbiota fueling an IL-6 driven, IL-17-dominant, systemic smoldering inflammatory milieu, ultimately leading to a vicious circle of obesity and inflammation. On the other hand, individuals consuming probiotic yogurt enrich their gut with probiotic bacteria that stimulate the anti-inflammatory arm of the immune system. Potent IL-10-associated Treg responses in these individuals rescue them from the inflammation-obesity cycle, thus increasing likelihood of a leaner physique.

To test whether probiotic-triggered Foxp3^+^ regulatory T cells were the key population and sufficient for reduced fat pathology in Western diet-fed mice, we used adoptive transfer of purified CD4^+^Foxp3^+^ Treg cells delivered by intraperitoneal injection into naïve syngeneic C57BL/6 *Rag2*-deficient mice. For these experiments, cell donors were green fluorescent protein (gfp)-Foxp3 transgenic C57BL/6 mice that had received *L. reuteri* in their drinking water, in addition to Western-style chow. We found significantly reduced abdominal fat pathology in recipients of *L. reuteri*-treated cells when compared with cells from donors eating Western diet alone (Fig. 4d). These adoptive transfer model data showed that probiotic-mediated protection from obesity resided in functions of Foxp3^+^ regulatory T cells. In line with these observations, selectively depleting Tregs by targeting CD25 [32] showed similar effects. We found eight-week-old Swiss mice eating *L. reuteri* and also simultaneously treated with anti-CD25 antibody rapidly developed morbid obesity, sluggish demeanor, and profound abdominal fat pathology during the ensuing three months (Fig. 5a); whereas control animals eating *L. reuteri* and treated with sham isotype matched IgG had reduced fat pathology and slender outcomes (Figs. 5a and 5b). Requirements for CD25^+^ cells for slenderizing benefits of *L. reuteri* were also evident in abdominal fat weights that were significantly increased after CD25 depletion (Fig. 5c). A role for CD25 in controlling inflammatory cytokines was displayed in peripheral blood in this model (Fig. 1d). Thus, dietary supplementation with *L. reuteri* seems to restore beneficial balanced Th17/Treg host immunity, even in individuals otherwise suffering from a pro-inflammatory immunity and chronic inflammation when dining on Westernized ‘fast food’-style diets (Fig. 6).

## Discussion

We devised an animal model to examine the mechanisms of diet [i.e., ‘fast food’ or ‘yogurt’] previously shown to impact obesity in humans [1]. Genetically outbred mice consuming ‘fast food’ mimicked adiposity patterns in people [1] and displayed enteric microbial similarities with other models and humans [11]. Mice eating yogurt – a food item most strongly linked with lean physique in human subjects [1] - were leaner than matched control animals. We demonstrate that a purified lactic acid microbe was sufficient to mediate this effect. Importantly, we show that 1) this lean outcome was achievable even while dining on Westernized chow, 2) eating probiotic microbes did not significantly change the established control or Westernized diet microbiome in stool, 3) *L reuteri* consumption did not significantly alter the caloric intake even though mice were significantly slimmer, and 4) lean outcomes involved microbe-triggered CD4^+^ Foxp3^+^ regulatory T cells, dependent on IL-10. These observations led us to propose a circular mechanistic model where both lactic acid organisms and the resident GI microbiome [1], [11] affect host immunity [21]–[22], which in turn affects obesity [31], which impact host immunity, and so forth. Once initiated, adiposity may then become a self-sustaining condition.

Consuming Westernized ‘fast food’-style chow rapidly re-structured the murine microbiome coincident with increased IL-17 protein levels and developing obesity in our mouse models [11]. In our human subjects, a Th17 biased profile also emerged when eating fast food, matching that seen in animals eating the Western chow. Although the New Western diet for mice was selected specifically to mimic human ‘fast food’, the ‘potato chip’ diet of humans also includes high salt and other factors not included in mouse chow. Nonetheless, these mice exhibited elevated levels of Il-17A, whether a result of the diet, or microbes, or from the obesity itself [6]. Following this line of reasoning, the insertion of dietary probiotics may break this inflammatory-adiposity cycle. Although a Th17 pro-inflammatory bias has been clearly linked with obesity, there’s evidence of a dichotomy in effects of IL-17A in adipogenesis [6], [33]–[34]. One possible explanation for this might be the recently uncovered heterogeneity amongst Th17 cells [35]–[37]. As a result, such complex immune feedback mechanisms are difficult to interrupt without untoward consequences to the host, making pro-inflammatory molecules TNF-α, IL-6 and IL-17 [8], [38]–[40] challenging targets in this process. These constraints are perhaps best overcome by directly harnessing bacteria that apparently induce global immune homeostasis that may serve public health initiatives.

In contrast with microbiome changes induced by eating ‘fast food’, consuming yogurt or purified probiotic bacteria in the form of *L. reuteri* did *not* significantly alter the existing gut microbiome as measured in stool in the present or other studies [41]. However, feeding of this lactic acid microbe *did* recapitulate leaner physique recently observed in a large epidemiological survey of human subjects when eating yogurt [1], and in line with recent work by others [42]–[47]. However, in separate epidemiological studies, the presence of *L. reuteri* in human guts has been associated with obesity [48]–[49]. Whether this discrepancy reflects the wide genomic variation of *L. reuteri* strains or differences [50] in host-microbial interactions warrants further investigation. A health-protective role for *L. reuteri* in host metabolism, as displayed in the present study, offers mutually beneficial gut symbiont-host relationship and co-evolution [51].

Interestingly, feeding probiotic yogurt or the purified *L. reuteri* bacteria to our mice did not alter their caloric intake, and also revealed benefits to general demeanor, skin and hair coat, and reproductive performance coincident with elevated plasma levels of oxytocin [52]. Oxytocin, a neuropeptide hormone associated with reproduction and social bonding, has also been linked with dietary satiety [53]–[54]. Hypothalamic hormones such as oxytocin conceptually intersect microbes with social and physical fitness in evolutionary success. Studies are underway to more explicitly test the impact of a microbial – hypothalamic – immune axis upon obesity.

We postulate that microbes contained in probiotic yogurt impart immune homeostasis that maintains systemic health [21]–[22]. One specific aspect of this paradigm is reciprocal activities of pro-inflammatory Th-17 and anti-inflammatory Treg cells [23]. Diet and microbe-induced failure of tolerance unifies these data with prior work involving inflammation, obesity, and cancer [8], [55]–[58]. These data agree with Feuerer et al (2009) who discovered IL-10-dependent Treg arise inversely with adiposity and insulin resistance [31]. Along mucosal surfaces, IL-10 facilitates immune tolerance [59] and recruitment of Treg cells to skew host immunity away from pro-inflammatory IL-17. Diverse disorders such as asthma and autoimmune diseases associated with Westernized living are widely believed to result from insufficient levels of IL-10 and insufficient immune calibration essential for sustained systemic health [60]. Westernized diets are also low in vitamin D, a nutrient that normally works together with IL-10 to enforce immune tolerance and protect against inflammatory disorders [61]–[63] and some types of cancer [64].

Feeding of palatable probiotic organisms offers potentially potent, cost-effective and practical options for public weight management. Apparent requirements for other beneficial resident commensal microbes may be further tested using gnotobiotic models [41], [65]–[66]. Such microbial re-programming may ultimately target other diseases linked with obesity and inflammation such as diabetes [2], cancer [8], and multiple sclerosis [15] for healthful longevity to combat a growing Westernized ‘fast food’ public health crisis.

## Experimental Procedures

### Animals

Genetically outbred CD-1 mice (Charles River; Wilmington, MA), inbred *wild type or Interleukin 10-deficient* C57BL/6 strain mice (Jackson Labs, Bar Harbor, ME), plus inbred *Rag2-deficient* C57BL/6 strain mice and outbred Swiss Webster mice (Taconic; Germantown, NY) were housed and handled in Association for Assessment and Accreditation of Laboratory Animal Care (AAALAC)-accredited facilities with diets, experimental methods, and housing as specifically approved by the Institutional Animal Care and Use Committee. The MIT CAC (IACUC) specifically approved the studies as well as the housing and handling of these animals. Mice were bred in-house to achieve experimental groups. The experimental design was to expose mice to diets starting at age = eight weeks, and then continue the treatment for 12 weeks until euthanasia using carbon dioxide at five months of age, unless otherwise specified (Fig. S1). Each experiment included 5–15 animals per group with two replications (total N = 10–30 mice per group). For microbiome analyses, fresh stools were collected twice weekly and stored in RNA-later at −20C for later testing. Other tissues were collected upon necropsy.

### Human Subjects and Specimen Collection

The PhenoGenetic Cohort of Brigham and Women’s Hospital including 1200 healthy control subjects provided subject recruitment and sample collection, in compliance with the Declaration of Helsinki with local ethics committee approval before initiation, and written informed consent from all subjects. The Brigham and Womens Institutional Review Board (IRB): Partners Human Research Committee specifically approved the studies. Subjects had the following characteristics: female/male sex ratio was 60∶40%; race distribution was 14% African American, 12% Asian American, 68% white, and 6% Hispanic; mean age was 24.3 y (range, 18–50 y); mean body mass index was 22.5 (range, 13–50). Blood samples were processed on the day of collection. Dietary and lifestyle behaviour was assessed by questionnaire on day of the visit. Total N = 23 for high fast food intake, and N = 26 for low fast food intake.

### Special Diets for Animals

Mice of 6–8 wks were placed on experimental diets: control AIN-76A (Harlan-Teklad Madison WI), and a Westernized diet with high fat and low fiber with substandard levels of Vitamin D (TD.96096; Harlan-Teklad) starting at 8 weeks of age until euthanasia at 5 months of age. Subgroups were supplemented with commercially available vanilla probiotic yogurt (0.8 ml/mouse 3X weekly) containing a microbial cocktail including *S. thermophilus, L. bulgaricus, L. acidophilus, Bifidobacterium bifidus, L. casei,* and *L. rhamnosus* during this same time. Separate groups of animals received a purified preparation of an anti-inflammatory strain of *Lactobacillus reuteri* ATCC 6475 cultivated as described elsewhere [24] using a dosage of 3.5×10^5^ organisms/mouse/day, or a sham *E coli* K12 3.5×10^5^ organisms/mouse/day in drinking water. Drinking water was replaced at least weekly. Viability of *L reuteri* organisms in drinking water was assessed using standard aerobic plate culture methods and determined to be too numerous to count (TNTC) at day one and less than ten colonies by day seven.

### Stool Microbiome Analyses

Genomic DNA was extracted from stool samples using the Qiagen QIAamp DNA Stool Mini Kit. Samples for paired-end Illumina sequencing were constructed using a two-step PCR amplicon approach targeting the V4 region of the 16S rRNA gene (U515F and E786R) as described in [67] and reads were quality filtered and clustered into operational taxonomic units (OTUs) at 97% nucleotide identity. Principal coordinates analysis is based on the Jensen-Shannon divergence between samples, and differences in microbiome composition were tested for significance using an empirical p-value estimated by permutation. Data presented in Fig. 1 were from stool collected at 5 days after onset of treatments.

### Human T Cell Isolation and Stimulation

Peripheral blood mononuclear cells (PBMC) were separated by Ficoll-Paque PLUS (GE Healthcare, Piscataway, NJ) gradient centrifugation. Untouched CD4^+^ T cells were isolated from PBMC by negative selection via the CD4^+^ T cell isolation kit II (Miltenyi Biotec, Auburn, CA). CD4^+^ T cells were cultured in serum-free X-Vivo 15 medium (BioWhittaker, Walkersville, MD) and stimulated for 4 h with PMA (50 ng/ml) and ionomycin (250 ng/ml; Sigma-Aldrich) with GolgiPlug (BD Biosciences). Stimulated cells, with dead cells excluded by LIVE/DEAD cell kit (Live Technologies), were fixed and made permeable according to manufacturer’s instructions (Fix/Perm; eBioscience) and were stained with anti-IL17A (eBio64DEC17; eBioscience, San Diego, CA) and anti-FOXP3 (206D; BioLegend, San Diego CA) for 30–45 min. Data were acquired on a LSR II (BD Biosciences, San Jose, CA) and analyzed with FlowJo software (TreeStart, Ashland OR) and GraphPad Prism (GraphPad Software, La Jolla, CA).

### Adoptive Transfer of T Cells into Recipient Mice

CD4^+^ lymphocytes isolated from *wild type or IL-10-deficient* C57BL/6 mice using magnetic beads (Dynal/Invitrogen; Carlsbad CA) are sorted by hi-speed flow cytometry (MoFlow2) to obtain purified populations of CD4^+^ lymphocytes and determined to be ∼96% pure as previously described elsewhere [16]. Syngeneic Rag2^−/−^ recipient mice were then injected intraperitoneally with 3×10^5^ CD4^+^ cells as previously described. For separate assays, purified populations of CD4^+^ lymphocytes were collected as above from transgenic C57BL/6 mouse donors expressing green fluorescent protein (gfp)-Foxp3, and then further purified sorting gfp^+^ expression to achieve purified populations of CD4^+^ FoxP3^+^ T_REG_ lymphocytes injected intraperitoneally at 3×10^5^ cells/mouse into C57BL/6 Rag2^−/−^ recipient mice.

### Depletion of CD25^+^ Cells

Mice were treated with anti-CD25 antibody (clone PC-61; Bio- Express, West Lebanon, NH) at 150 ug per mouse intraperitoneally 3X weekly for 12 weeks. Treated mice were compared to mice receiving sham isotype antibody alone. Depletion of CD25^+^ cells was confirmed by undetectably low fractions of CD25+ cells in spleens of mice treated with anti-CD25 antibody compared to sham-treated controls using flow cytometry. Depletion was confirmed by absence of Foxp3^+^ cells in spleen.

### Detection of Systemic Cytokine Protein Expression

Serum cytokine levels of six animals per group were analyzed using the Bioplex assay system (BioRad, Hercules, CA) according to the manufacturers protocol. Samples were analyzed in duplicate on a Bio-Plex 200 system (BioRad, Hercules, CA). Statistical analysis was performed using 2-tailed student’s t-test; a p-value <0.05 was considered statistically significant.

### Histopathology and Immunohistochemistry

For histologic evaluation, formalin-fixed tissues were embedded in paraffin, cut at 5 µm, and stained with hematoxylin and eosin. Lesions were analyzed and quantified by a pathologist blinded to sample identity. Foxp3 and Il-17 were labeled immunohistochemically in mouse tissue. Immunohistochemistry and morphometric assessment of Il-17^+^ and FoxP3^+^ cells in mesenteric lymph nodes and abdominal fat were as previously described [16].

### Statistical Analyses

The Mann-Whitney U test was used for body weight, diet, calorie consumption, and histomorphometry. A p-value <0.05 was statistically significant.

## Supporting Information

Figure S1 **Dietary probiotic bacteria protect mice from obesity.** The experimental time line depicts how outbred Swiss mice or inbred C57BL/6 mice began eating special diets at 8-weeks-of-age. *Ad libitum* diets were fed continuously for three months duration until mice were humanely euthanized at 5-months-of-age **(a)**. A significantly slender body weight effect was achieved by adding 3×10^5^ *L reuteri* organisms/mouse/day to drinking water, but similar addition of 3×10^5^ *E. coli* K12 organisms/mouse/day to drinking water did not cause significant differences when compared with untreated controls **(b)**. Female Swiss mice eating either probiotic yogurt or purified *L. reuteri* organisms have significantly lower body weights than their non-probiotic-fed counterparts. Data are shown in 5-month-old (yogurt consuming) and 9-month-old (aged *L reuteri*-consuming) mice **(c)**. Similarly to what has been observed in male mice, probiotics protect female mice from abdominal fat pathology, upregulate Foxp3+ cells in the MLNs and downregulate IL-17 expression in the abdominal fat and the MLNs **(d)**. CLS in the abdominal fat of obese male and female mice are often characterized by a robust inflammatory response with high numbers of macrophages, lymphocytes, neutrophils and myeloid precursor cells. In contrast, CLS of probiotic-fed mice maintain their typical quiescent inflammatory lesion appearance with macrophages and occasional lymphocytes bordering dead adipocytes **(e)**. IL-17 specific immunohistochemistry shows the abundant cytoplasmic and extracellular IL-17 found in the MLNs of western-diet fed obese Swiss mice but not in the MLNs of mice consuming the same diet plus probiotics **(f)**. Obesity-associated adipose tissue pathology was noticed in all fat depots of the mouse body examined including the mesenteric fat shown here. Probiotics universally suppressed this pathology **(g)**. Experiments in germ-free mice suggest that diverse bacteria are required for slim outcomes since these mice did not benefit after eating probiotics **(h)**. p<0.05, ** p<0.001, *** p<0.0001. Hematoxylin and eosin (c and e); Diaminobenzidine chromogen, hematoxylin counterstain (d). Bars = 25 µm (c and d); 100 µm (e).
(TIF)
