# Supplementary figures and images for: Microbial Reprogramming Inhibits Western Diet-Associated Obesity
Source: PLoS One. 2013 Jul 10;8(7):e68596. doi: 10.1371/journal.pone.0068596 (PMC3707834; doi:10.1371/journal.pone.0068596)

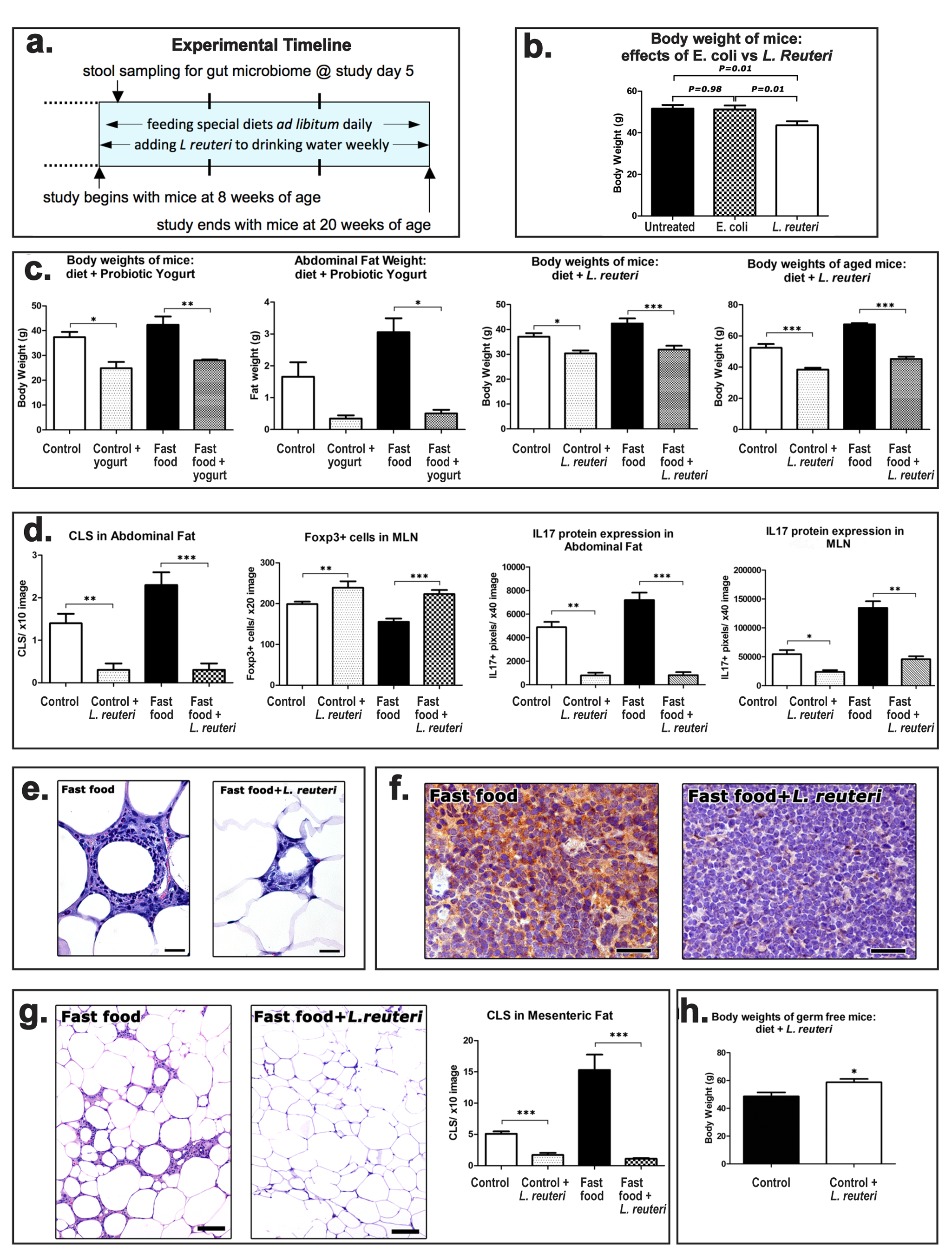

Supplement: Figure S1 — Dietary probiotic bacteria protect mice from obesity. The experimental time line depicts how outbred Swiss mice or inbred C57BL/6 mice began eating special diets at 8-weeks-of-age. Ad libitum diets were fed continuously for three months duration until mice were humanely euthanized at 5-months-of-age (a). A significantly slender body weight effect was achieved by adding 3×10^5^ L reuteri organisms/mouse/day to drinking water, but similar addition of 3×10^5^ E. coli K12 organisms/mouse/day to drinking water did not cause significant differences when compared with untreated controls (b). Female Swiss mice eating either probiotic yogurt or purified L. reuteri organisms have significantly lower body weights than their non-probiotic-fed counterparts. Data are shown in 5-month-old (yogurt consuming) and 9-month-old (aged L reuteri-consuming) mice (c). Similarly to what has been observed in male mice, probiotics protect female mice from abdominal fat pathology, upregulate Foxp3+ cells in the MLNs and downregulate IL-17 expression in the abdominal fat and the MLNs (d). CLS in the abdominal fat of obese male and female mice are often characterized by a robust inflammatory response with high numbers of macrophages, lymphocytes, neutrophils and myeloid precursor cells. In contrast, CLS of probiotic-fed mice maintain their typical quiescent inflammatory lesion appearance with macrophages and occasional lymphocytes bordering dead adipocytes (e). IL-17 specific immunohistochemistry shows the abundant cytoplasmic and extracellular IL-17 found in the MLNs of western-diet fed obese Swiss mice but not in the MLNs of mice consuming the same diet plus probiotics (f). Obesity-associated adipose tissue pathology was noticed in all fat depots of the mouse body examined including the mesenteric fat shown here. Probiotics universally suppressed this pathology (g). Experiments in germ-free mice suggest that diverse bacteria are required for slim outcomes since these mice did not benefit after eat [file pone.0068596.s001.tif]
